# Editorial: Honesty and Moral Behavior in Economic Games

**DOI:** 10.3389/fpsyg.2021.769856

**Published:** 2021-10-04

**Authors:** Steffen Huck, Agne Kajackaite, Nora Szech

**Affiliations:** ^1^Social Science Research Center Berlin, Berlin, Germany; ^2^University College London, London, United Kingdom; ^3^Chair of Political Economy, Karlsruhe Institute of Technology (KIT), Karlsruhe, Germany

**Keywords:** morality, lying, honesty, ethics, economic games, behavioral economics, experiments

## Research on Moral Behavior in Behavioral Economics

It is in crisis times that we can see the ailments of society under a magnifying glass and all three major crises we have recently been facing and continue to face, the financial crisis of 2007 and its aftermath, the current Corona crisis, and the climate crisis that will only ever become worse, have put a spotlight on greedy and dishonest behavior which needs to be tackled if societies want to escape such ordeals half-way unscathed. Yet politicians can simply ignore key problems in their campaigns to get more votes; decision makers can get involved in corrupt behaviors for monetary benefits; and ordinary citizens can simply close their eyes trying to justify selfish acts –fueling crises further. It is, thus, not surprising that immoral behaviors and their root causes have received increasing attention in the last decade of the social science literature. In this collection we present 11 exciting new studies exploring the morality of behavior from the vantage point of (behavioral) economics.

From a standard economic perspective, the decision to behave immorally for a monetary benefit is affected by only two factors—the probability of being caught and the penalty resulting from it (see Becker, 1968). However, the fast-growing literature in behavioral economics shows that many people would forego an immoral action, such as lying, even if there is no possibility of being caught and being punished (see, for instance, Gneezy, 2005; Mazar et al., 2008; Shalvi et al., 2011; Fischbacher and Föllmi-Heusi, 2013; Abeler et al., 2014, 2019; Gächter and Schulz, 2016; Kajackaite and Gneezy, 2017; Gneezy et al., 2018). These studies show that some people lie only partially or do not lie at all, because they have an intrinsic cost of lying (a self-image cost) and/or because they do not want to be perceived as liars by others or themselves (image concerns). Another stream of research shows that moral behavior can be eroded in market interactions and voting (see, for instance, Falk and Szech, 2013; Bartling et al., 2015, Falk et al., 2020; Ziegler et al., 2020), and that the psychological cost of immoral behavior can be reduced by choosing to be ignorant about the consequences of one's, actions on others (see Dana et al., 2007; Exley, 2015; Grossman and van der Weele, 2017; Serra-Garcia and Szech, 2021).

These papers are just the tip of the iceberg—indeed morality has become one of the most popular topics in behavioral economics, and we are learning many valuable lessons about the forces influencing people's choices to behave in a more or less moral or honest way. With this Research Topic, we contribute to the literature by shedding more light on mechanisms that drive morally relevant behaviors.

## This Research Topic

This Research Topic consists of 11 research papers, with each of them using lab or field experiments to answer their research questions. The content of the contributions, forming this special issue, ranges from contributions on lying behavior (contributions by Behnk and Reuben; Dunaiev and Khadjavi; Jacquemet et al.; Vorsatz et al.; Waeber), bribing (contributions by Balafoutas et al.; Wang and Chen), pro-sociality (contributions by Regner; Regner and Matthey), and discrimination (contribution by Feess et al.) up to an experiment aiming to reduce meat consumption (contribution by Haile et al.). Taking a wholistic viewpoint as in Bandura (2016), morally relevant behavior may include caring about nature, the environment, and animals as well. A reduction in meat consumption may help us tackle the climate crisis. More broadly, as in the current Corona crisis, fostering morally relevant behaviors will hopefully contribute to dealing with the fallout from major crises, ideally helping to overcome them successfully.

## Future Directions

As demonstrated by this special issue, behavioral economics of morality is a fruitful field of research. While the topic is slowly maturing, the scope for future studies remains large with many important understudied applications. One of these is science itself including, as we were learning while writing this introduction, the very subfield this issue deals with. While it is tempting to dwell on the irony it is probably more interesting and clearly more important to understand the mechanisms that enable fraudulent behavior in the sciences. The case is complicated partially because of academia's self-government. When immoral behavior can only be verified by a select few, the question easily becomes who observes the observer? And what are the observer's interests? Of course, universities do not like scandals. But sweeping things under the carpet may ultimately be the more dangerous strategy. There is a lot to be worked on.

## Author Contributions

All authors listed have made a substantial, direct and intellectual contribution to the work, and approved it for publication.

## Conflict of Interest

The authors declare that the research was conducted in the absence of any commercial or financial relationships that could be construed as a potential conflict of interest.

## Publisher's Note

All claims expressed in this article are solely those of the authors and do not necessarily represent those of their affiliated organizations, or those of the publisher, the editors and the reviewers. Any product that may be evaluated in this article, or claim that may be made by its manufacturer, is not guaranteed or endorsed by the publisher.

## References

[B1] Abeler J. Becker A. Falk A. (2014). Representative evidence on lying costs. J. Public Econ. 113, 96–104. 10.1016/j.jpubeco.2014.01.005

[B2] Abeler J. Nosenzo D. Raymond C. (2019). Preferences for truth-telling. Econometrica 87, 1115–1153. 10.3982/ECTA14673

[B3] Bandura A. (2016). Moral Disengagement: How People Do Harm and Live With Themselves. New York, NY: Macmillan, 544.

[B4] Bartling B. Weber R. Yao L. (2015). Do markets erode social responsibility? Q. J. Econ. 130, 219–266. 10.1093/qje/qju031

[B5] Becker G. S. (1968). Crime and punishment: an economic approach. J. Polit. Econ. 76, 169–217. 10.1086/259394

[B6] Dana J. Weber R. A. Kuang J. X. (2007). Exploiting moral wiggle room: experiments demonstrating an illusory preference for fairness. Econ. Theory 33, 67–80. 10.1007/s00199-006-0153-z

[B7] Exley C. (2015). Excusing selfishness in charitable giving: the role of risk. Rev. Econ. Stud. 83, 587–628. 10.1093/restud/rdv051

[B8] Falk A. Neuber T. Szech N. (2020). Diffusion of being pivotal and immoral outcomes. Rev. Econ. Stud. 87, 2205–2229. 10.1093/restud/rdz064

[B9] Falk A. Szech N. (2013). Morals and markets. Science 340, 707–711. 10.1126/science.1231566 23661753

[B10] Fischbacher U. Föllmi-Heusi F. (2013). Lies in disguise—an experimental study on cheating. J. Eur. Econ. Assoc. 11, 525–547. 10.1111/jeea.12014

[B11] Gächter S. Schulz J. F. (2016). Intrinsic honesty and the prevalence of rule violations across societies. Nature 531, 496–499. 10.1038/nature17160 26958830 PMC4817241

[B12] Gneezy U. (2005). Deception: the role of consequences. Am. Econ. Rev. 95, 384–394. 10.1257/0002828053828662

[B13] Gneezy U. Kajackaite A. Sobel J. (2018). Lying aversion and the size of the lie. Am. Econ. Rev. 108, 419–453. 10.1257/aer.20161553

[B14] Grossman Z. van der Weele (2017). Self-image and willful ignorance in social decisions. J. Eur. Econ. Assoc. 15, 173–217. 10.1093/jeea/jvw001

[B15] Kajackaite A. Gneezy U. (2017). Incentives and cheating. Games Econ. Behav. 102, 433–444. 10.1016/j.geb.2017.01.015

[B16] Mazar N. Amir O. Ariely D. (2008). The dishonesty of honest people: a theory of self-concept maintenance. J. Mark. Res. 45, 633–644. 10.1509/jmkr.45.6.633

[B17] Serra-Garcia M. Szech N. (2021). The (In)Elasticity of moral ignorance. Manag. Sci. Available online at: https://www.cesifo.org/en/publikationen/2019/working-paper/inelasticity-moral-ignorance

[B18] Shalvi S. Dana J. Handgraaf M. J. J. De Dreu C. K. W. (2011). Justified ethicality: observing desired counterfactuals modifies ethical perceptions and behavior. Organ. Behav. Hum. Decis. Process. 115, 181–190. 10.1016/j.obhdp.2011.02.001

[B19] Ziegler A. Romagnoli G. Offerman T. (2020). Morals in multi-unit markets. Tinbergen Institute Discussion Paper 2020-072/I. Amsterdam. Available online at: https://ideas.repec.org/p/tin/wpaper/20200072.html

